# Ammonia Oxidizing Prokaryotes Respond Differently to Fertilization and Termination Methods in Common Oat’s Rhizosphere

**DOI:** 10.3389/fmicb.2021.746524

**Published:** 2021-10-06

**Authors:** Marco Allegrini, Marianela E. Morales, Maria B. Villamil, María Celina Zabaloy

**Affiliations:** ^1^Instituto de Investigaciones en Ciencias Agrarias de Rosario (IICAR), CONICET, Universidad Nacional de Rosario, Zavalla, Argentina; ^2^Centro de Recursos Naturales Renovables de la Zona Semiárida (CERZOS), Universidad Nacional del Sur (UNS)-CONICET, Bahía Blanca, Argentina; ^3^Department of Crop Sciences, University of Illinois, Urbana, IL, United States; ^4^Departamento de Agronomía, Universidad Nacional del Sur, Bahía Blanca, Argentina

**Keywords:** *amoA*, inorganic nitrogen, glyphosate, cover crop management, *Avena sativa* L

## Abstract

Cover crops (CC) have demonstrated beneficial effects on several soil properties yet questions remain regarding their effects on soil microbial communities. Among them, ammonia-oxidizing bacteria (AOB) and ammonia-oxidizing archaea (AOA) have a key role for N cycling in soil and their responses in the rhizosphere of terminated CC deserve further investigation. A greenhouse experiment was established to assess N fertilization (with or without N) and termination methods (glyphosate, mowing, and untreated control) of common oat (*Avena sativa* L.) as potential drivers of AOA and AOB responses in the rhizosphere. The abundance of *amoA* genes was determined by quantitative real-time PCR (qPCR), the community structure was assessed with Illumina amplicon sequencing of these genes, while the function was assessed from potential nitrification activity (PNA). While N fertilization had no influence on AOA, the termination method significantly increased *amoA* gene copies of AOA in mowed plants relative to glyphosate termination or the untreated control (1.76 and 1.49-fold change, respectively), and shifted AOA community structure (PERMANOVA, *p*<0.05). Ordination methods indicated a separation between AOA communities from control and glyphosate-terminated plants relative to mowed plants for both UniFrac and Aitchison distance. Converserly, N fertilization significantly increased AOB abundance in the rhizosphere of mowed and control plants, yet not in glyphosate-treated plants. Analyses of community structure showed that AOB changed only in response to N fertilization and not to the termination method. In line with these results, significantly higher PNA values were measured in all fertilized samples, regardless of the termination methods. Overall, the results of this study indicated that bacterial and archaeal nitrifiers have contrasting responses to fertlization and plant termination methods. While AOA were responsive to the termination method, AOB were more sensitive to N additions, although, the stimulative effect of N fertilization on *amoA*_AOB_ abundance was dependent on the termination method.

## Introduction

One of the biggest challenges of modern agriculture is the production of enough high-quality food, while reducing the environmental impact and the dependence on external inputs. In this context, cover crops (CCs) have gained popularity as a sustainable alternative with demonstrated beneficial effects on several soil properties (Lu et al., 2000; Chavarría et al., 2016). CCs are defined as crops grown between periods of normal crop production as an alternative to bare soil in fallow months, to provide soil protection and soil improvement (Soil Science Society of America, 1997). The termination of the CC growth is required to allow planting of the cash crop and includes both mechanical (e.g., rolling or mowing) and chemical methods (Lu et al., 2000). In no-tillage crop production, chemical killing by herbicides is the conventional approach and the broad spectrum and non-selective herbicide glyphosate (N-[phosphonomethyl]glycine) is one of the most used herbicides to achieve this purpose (Fageria et al., 2005). To fully assess the contribution of CC to sustainable agriculture, a broader and deeper view is required considering the several factors involved in this agricultural practice. In particular, how CC management practices affect soil microbial communities and their functions has been comparatively less studied than the influence of more traditional agricultural practices on soil microorganisms.

Several studies have investigated the effects of CC on soil microbiology, reporting increases in soil microbial biomass (King and Hofmockel, 2017), evenness of bacterial taxa (Li et al., 2012), and microbial enzymatic activities (Chavarría et al., 2016). A recent meta-analysis by Kim et al. (2020) covering 60 studies globally, reported that CC significantly increased soil microbial abundance, activity and diversity. At the same time, the study concluded that CC effect sizes varied by agricultural covariates such as the CC termination method. The latter is particularly relevant during the post-CC termination period as CC and their residues, including roots and their associated microbial communities, are keystones in the regulation of nutrient availability for the subsequent cash crops through decomposition processes (Frank and Groffman, 2009; Sievers and Cook, 2018).

The effect of the termination method and its interaction with fertilizers have never been explored in the rhizosphere of a CC. Although, CC are used to capture leachable nitrates in the soil, non-leguminous CC are usually supplied with “starter” N fertilizer due to their high response to external N inputs (Ridley, 2012). After CC termination, above and below-ground plant material starts decomposing. Cash crops planted at or around CC termination will be influenced by the N pool in the soil resulting from both ammonification and ammonia-oxidation processes (Kuo et al., 1997; Sievers and Cook, 2018). Thus, the differences in the structure and activity of microbial communities between mechanically suppressed and chemically desiccated CC could translate into differences in N availability for the following crop. This study is focused on functionally specialized groups that have a key role in the N cycle such as ammonia-oxidizing bacteria (AOB) and ammonia-oxidizing archaea (AOA). Ammonia oxidizers perform the first and rate-limiting step of nitrification, through the oxidation of ammonia derived from both inorganic fertilization and the mineralization of organic matter (Levičnik-Höfferle et al., 2012; Lu et al., 2020). These nitrifiers exhibit niche and physiological differentiation: while low ammonium supply from mineralization of organic matter favors growth and activity of AOA, AOB prefer high ammonium supply from inorganic fertilizer inputs (Stopnišek et al., 2010; Levičnik-Höfferle et al., 2012). Hink et al. (2018) demonstrated that AOA successfully outcompete AOB when NH_4_^+^ is continuously supplied at a low rate in the soil through mineralization of native organic N, or when a slow-release fertilizer is used to supplement. Although, recent studies have provided evidences of the contribution of complete ammonia oxidizers (comammox) to nitrification in agricultural soils (Pjevac et al., 2017; Orellana et al., 2018; Wang et al., 2019), we restricted our study to AOA and AOB as the most widely studied ammonia oxidizers in the rhizosphere.

Previous studies have focused on AOA and AOB as sensitive microbial indicators to disturbances (Ritz et al., 2009; Wessén and Hallin, 2011; Pereira e Silva et al., 2012), management practices (Bru et al., 2010), and even glyphosate applications (Zhang et al., 2018). Fewer studies have examined the effects of CC termination on nitrifying prokaryotes (Caliz et al., 2015; Romdhane et al., 2019). In a previous greenhouse study of CC termination methods, Allegrini et al. (2019) found a lower abundance of AOB and AOA in the rhizosphere of unfertilized common oat terminated with glyphosate compared to mechanical termination, yet activity, and diversity of ammonia oxidizers were not assessed. The authors also reported differences in the catabolic profiles of microbial communities between termination methods, most likely due to specific rhizodeposition patterns and root turnover rates in mowed and glyphosate-treated plants. Differences in the quality and quantity of root exudates between glyphosate-treated and control plants have been reported in several studies (Kremer et al., 2005). Glyphosate treated plants showed higher exudation of amino acids and carbohydrates relative to mowed plants, and faster necrosis of root tissue was observed at the microscopic level (Imparato et al., 2016).

Thus, based on the previous literature, we hypothesized that due to their described effects on rhizodeposition and root turnover, termination methods will ultimately affect AOB and AOA (alpha and beta diversity of communities, abundance, and activity) relative to the untreated controls, regardless of the fertilization treatment. We also hypothesized that inorganic N fertilization will favor AOB over AOA in the rhizosphere of oat, regardless of the termination method. The overall objective of this study was to assess the effects of CC termination methods and N fertilization as potential drivers of the prokaryotic ammonia oxidizers’ activity and diversity in the rhizosphere. The results will contribute to further our understanding of how the prokaryotic ammonia oxidizers respond to management strategies that are critical in sustainable agriculture.

## Materials and Methods

### Experimental Design

In March 2018, a composite soil sample (0–20cm) was collected from an experimental site at the Universidad Nacional del Sur Campus (38°41.64′ S, 62°14.46′ W) in Bahía Blanca (Argentina), to fill 5.5dm^3^ pots, each with 5.6kg of soil. The sample belongs to a well-drained Petrocalcic Paleustoll with 15years of *A. sativa* L. cropping history. Aldana and Carioni (2001) reported the following properties for this soil: sandy loam texture and bulk density 1.28gcm^−3^, while a soil analysis in 2018 indicated a pH_(H2O)_ 7.6 (1:2.5 soil:water), organic matter 2.46%, extractable (Bray) P 7.1mgkg^−1^, N-NH_4_^+^ 13.3mgkg^−1^, N-NO_3_^−^ 9.1mgkg^−1^ and of total N 0.129%.

The greenhouse assay was conducted with *A. sativa* L. var. Cristal INTA. The experimental factors were fertilization level (with and without inorganic N fertilizer) and CC termination method (M: mowing, G: glyphosate, and U: untreated control) arranged as a 2 × 3 factorial in a completely randomized design, resulting in a total of six treatment–combinations, with four replicates (pots) per treatment. For glyphosate termination, the herbicide was applied as a commercial formulation (3lha^−1^, Eskoba Full II, Red Surcos, 662gl^−1^, monopotassium salt) using a knapsack sprayer. The total growth period from CC planting (April 3) to termination date was 92days (Z3.1 stage) with automatized irrigation twice a day during 5min through sprinklers. The N fertilizer (urea, 46% N) was surface applied at planting and tillering stage (day 76) at a total rate of 100kgNha^−1^. Untreated control pots were destructively sampled at the time of termination to collect rhizospheric soil of living roots. For the experimental units terminated with glyphosate and mowing, the soil was sampled 12days later, to collect rhizospheric soil of plants with notable desiccation symptoms. In all cases, loosely adhering soil was removed by gentle shaking of the root system to discard bulk soil, and the tightly adhering soil (rhizospheric soil) was removed by brushing the root system with sterile brushes, collected on sterile trays, and stored in sterile plastic bags (Yanai et al., 2003). The soil was stored at −80°C for DNA extraction and molecular analysis and at 4°C for assessment of PNA.

### Potential Nitrification Activity

Nitrification rate was measured according to the potential nitrification assay described by Hart et al. (1994), with modifications in the slurry preparation (10-fold scaling, i.e., 1.5g of fresh soil in 10ml of reaction buffer) to adapt volumes and soil quantities to a microplate assay, similar to Hoffmann et al. (2007). PNA reaction buffer (0.3mM KH_2_PO_4_, 0.7mM K_2_HPO_4_, 0.05mM (NH_4_)_2_SO_4_, and 10mM KClO_3_) was freshly prepared on the same day in which soil slurries were incubated. The slurries were prepared in 125ml sterile flasks loosely capped with aluminum foil to reduce water evaporation while leaving enough space for aeration. Flasks were incubated in an orbital shaker (25°C, 180rpm in the dark) for 1h to achieve a homogeneous suspension, and, afterward, 1ml samples were taken at 0, 2, 4, 20, and 22h (Hart et al., 1994). Samples were centrifuged at 15,000rpm (RCF: 21,379×*g*, 4°C) in a Hermle Z32-HK microcentrifuge (Wehingen, Germany) and the supernatant was transferred to sterile 1.5ml tubes. The tubes were immediately frozen at −20°C.

Quantitative analysis of nitrite concentration was conducted in 96-wells, polypropylene, flat-bottom microplates (Eppendorf AG, Hamburg, Germany, reference number: 0030 602.102) according to the diazotization method (Griess reaction) with sulfanilic acid (0.8% w/v in acetic acid; reference number: B1550261, Laboratorios Britania S.A., Buenos Aires, Argentina) and N-(1-Naphthyl)ethylenediamine 2HCl 0.1% w/v in acetic acid (Carbosynth^TM^, Compton, United Kingdom). Absorbance was measured at 540nm and 25°C in a FLUOstar Optima microplate reader (BMG Labtech, Offenburg, Germany). The rate of production of nitrite was calculated by linear regression of solution concentration over time. After correction for soil moisture content of the soil and conversion to a soil dry weight (dw) basis, the potential nitrification activity (μgN-NO_2_^−^ g^−1^ dw soil h^−1^) was calculated following Drury et al. (2006).

### Assessment of Potential Nitrification Activity With Root Exudates

The collection of root exudates was done from mowed or glyphosate treated plants (unfertilized) with a solution of CaCl_2_, as described by Egle et al. (2003). Briefly, all mineral particles attached to the root system in each pot were removed with tap water and the roots were submerged in 100ml of collection solution (CaCl_2_ 0.05mM, pH 5.5) for 1h. The liquid was discarded and the roots were submerged again in 50ml of the collection solution for 16h. The resulting exudate obtained in each case was centrifuged 10min at 13,000×g, filter-sterilized, lyophilized, and stored at −20°C. The lyophilized exudate from each pot was suspended in 10ml of PNA reaction buffer and immediately used to assess the effect on ammonia oxidation. Two pools of root exudates (one from glyphosate-terminated plants and one from mowed plants) were obtained by mixing equal quantities of exudates from each pot of the same treatment group.

To assess the effect of root exudates on nitrifying communities, soil samples (approximately 1.5g) from each of the four replicates (pots) collected at time zero (before termination) were divided into three portions (each about 0.4g) to prepare soil slurries with either root exudates collected from glyphosate-treated plants, root exudates collected from mowed plants, or soil slurries with the PNA reaction buffer (control). The reaction buffer and the conditions of incubation were as described previously except for the sampling times (0, 2, 20, 22, and 24h). Soil slurries were prepared by combining the soil samples with 9ml of reaction buffer and 1ml of root exudate, or soil samples with 10ml of PNA reaction buffer for the control flasks.

### DNA Extraction and Quantification

DNA was extracted from 250mg of rhizospheric soil samples with the commercial kit PowerSoil DNA Isolation kit (Qiagen, Hilden, Germany) according to manufacturer instructions. DNA was quantified using QuantiFluor dsDNA kit in a Quantus fluorometer (Promega, Promega, Madison, WI, United States). The quality of the DNA was assessed from 1% agarose gel and absorbance ratios (260:230 and 260:280nm ratio) in a DS-11 FX spectrophotometer (DeNovix Inc., Wilmington, DE, United States).

### Quantitative PCR of *amoA* Gene

To study the treatment effects on the estimated abundance of AOB and AOA, a quantitative real-time PCR (qPCR) of *amoA* gene of each group was conducted (*amoA*_AOB_ and *amoA*_AOA_, respectively). Primers used for AOA and AOB as well as their amplicon lengths are indicated in Supplementary Table S1. To avoid introducing errors (mainly related to an unknown number of operons per cell), we analyzed the copy numbers (Ouyang et al., 2016; Morrison et al., 2017); no attempt was made to convert these copies into cell numbers. The amplification program for AOB and AOA as well as the PCR master mix volumes and reaction setup were previously reported by Zabaloy et al. (2016) and Zabaloy et al. (2017), respectively. All amplifications, baseline corrections, melting curve analysis, and standard curve assessments were conducted in ABI 7500 Real−Time System and its associated software (7500 Software v2.0.3, Applied Biosystems, Foster City, CA). The equation of the standard curve for *amoA*_AOB_ (Ct=39.084–3.881 log_10_ copy number) represented an assay efficiency of 81% with an *R*^2^ value of 0.993, while the equation for *amoA*_AOA_ (Ct=33.55–3.76 log_10_ copy number) represented an efficiency of 84% with an *R*^2^ value of 0.996.

### Amplicon Sequencing of *amoA* Gene

Three replicates of each treatment were analyzed by amplicon sequencing of *amoA* gene. Gene libraries were prepared with the primers amoA1F/amoA2R (*amoA*_AOB_) and CrenamoA23f/CrenamoA616r (*amoA*_AOA_; Supplementary Table S1), using the Fluidigm™ protocol at the DNA Service Laboratory, Roy J. Carver Biotechnology Center at University of Illinois (Urbana-Champaign, United States). The library was quantitated by qPCR and sequenced on an Illumina MiSeq platform (Illumina, San Diego, CA, United States) using one MiSeq flowcell for 251cycles, from each end of the fragments. FASTQ files were generated and demultiplexed by the sequencing service with the bcl2fastq v2.20 Conversion Software (Illumina, San Diego, CA, United States).

### Bioinformatic Analysis of Amplicon Sequencing Data

The FASTQ files were processed in QIIME2 (Bolyen et al., 2019) using the recommended pipelines for paired-end reads (AOB) or single-end reads (AOA) based on DADA2 denoising algorithm. Briefly, primers were removed using *p-trim-left* argument within *dada2 denoised-paired* script (AOB) or within *dada2 denoised-single* script (AOA). For AOA, we used *dada2 denoised-single* script (i.e., only forward reads) considering that the length of the reads (300bp) was not enough for merging forward and reverse reads of the amplicon and, thus, a pipeline for paired reads would be inappropriate in this case. The trimming argument within *dada2 denoised-paired* script was set on 273bp for forward reads and 240 for reverse reads (median Q values >27 reported by Interactive Quality Plot tool of QIIME2), while a default value of 2 was set for the expected number of errors. For *dada2 denoised-single*, script we used the same expected number of errors, while no trimming was applied as the quality values were acceptable (median Q values >33). Single-ends analysis yields sequences that are inherently shorter than in paired-end analysis, thus a trimming step would shorten the length of the amplicon even further for taxonomic analysis.

The resulting amplicon sequence variants (ASVs) were tabulated and the ASV table was rarefied to a value equal to the sum of reads of the sample with the lower number of reads. The rarefied data of ASV table was used in vegan package 2.5–7 (Oksanen et al., 2020) of R Statistical Software v4.1.0 (R Core Team, 2021) for calculation of the following alpha diversity metrics: Shannon diversity index (H′), observed richness (S′=number of ASVs), and Pielou’s evenness index (J’). The ASVs obtained after all processing steps (removal of primers, trimming and filtering, denoising, and chimera removal) were clustered into operative taxonomic units (OTUs) using *qiime vsearch cluster-features-de-novo* at the appropriate species-level identity threshold for *amoA* gene (90%; Ouyang et al., 2016), similarly to other studies (Ouyang et al., 2016; Lourenço et al., 2018). For calculation of UniFrac distances, the representative sequences of the resulting OTUs were then aligned using Muscle algorithm in MEGAX (Kumar et al., 2018) and the aligned sequences were loaded in phangorn v2.7.0 package (Schliep, 2011) of R Statistical Software v.4.1.0 to obtain the distance matrix and a neighbor-joining tree. The likelihood of this tree was computed (*pml function*) and then optimized (*optim.pml*) using the most appropriate model of nucleotide evolution according to the Bayesian Information Criterion (BIC; Schliep, 2018). The phylogenetic tree and the OTU table were used in GUniFrac package v1.2 (Chen, 2021) of R Statistical Software v.4.1.0 to calculate generalized UniFrac distances.

Phylogenetic trees containing the OTU sequences and database sequences of *amoA* gene of AOB and AOA were constructed using maximum likelihood method with 1,000 bootstraps in MEGAX (Kumar et al., 2018). Tamura 3-parameter model and Hasegawa-Kishino-Yano model were used for AOA and AOB, respectively, according to the lowest Bayesian Information Criterion of model selection. We used high-quality *amoA* sequences from FunGene database (Fish et al., 2013) also included by Lourenço et al. (2018), with a score above 350, a size greater than 200 amino acids in length, a hidden Markov model (HMM) coverage of more than 85%, and a defined organism. Additional sequences from members of *Nitrosospira* clusters 0, 2, and 4, also included in reported AOB phylogenetic trees of rhizospheric soil (Glaser et al., 2010), were included as well. For AOA, we used several *amoA* sequences from marine environment, soil, sediments, and the rhizosphere of *Zea mays* L., all previously used in the construction of phylogenetic trees of AOA (Park et al., 2006; He et al., 2007; Tourna et al., 2011; Yao et al., 2011; Ai et al., 2013). Interactive Tree of Life (iTOL) tool was used for tree visualization (Letunic and Bork, 2016).

### Statistical Analysis

All statistical analyses were conducted in R Statistical Software v.4.1.0 (R Core Team, 2021). Quantitative PCR data (copy number μg^−1^ DNA) and PNA values were analyzed using two-way ANOVA and least-square means procedure for *post hoc* multiple comparisons of means (*α*=0.05) after a log_10_ transformation in emmeans package v.1.6.1 (Lenth, 2021). The interaction term was considered significant at *p*<0.2 (Littell et al., 2002). The alpha-diversity metrics were analyzed using two-way ANOVA (*α*=0.05) with the rarefied ASV table. The PNA in response to the addition of root exudates was analyzed using a linear mixed model in nlme package v3.1–152 (Pinheiro et al., 2021) considering the pot (replicate) from which soil was originally extracted as a random effect, and the treatment (glyphosate root exudate, mowing root exudate, or control buffer) as a fixed effect. Correlation analyses between the copy number of *amoA* genes and the PNA were performed using Pearson method (*α*=0.05) with *cor.test* function in base R.

Amplicon sequencing data was analyzed using both the standard approach as well as the compositional approach (Gloor and Reid, 2016; Gloor et al., 2017) using several packages of R Statistical Software v.4.1.0. In the first case, the UniFrac distances were used as input in vegan package v2.5–7 (Oksanen et al., 2020) for multivariate analysis of beta diversity through non-metric multidimensional scaling (NMDS) using *metaMDS* function. In the second case, centered log-ratio (clr) transformed data (Aitchison, 1986) was first calculated after replacing zero values with zCompositions package v.1.3.4 (Palarea-Albaladejo and Martin-Fernandez, 2015) and then used as input for principal component analysis (PCA) with *PCA function* (singular value decomposition) using FactoMineR package v.2.4 (Lê et al., 2008). The covariance biplots of compositional data were obtained using the packages FactoMineR, factoextra v.1.0.7 (Kassambara and Mundt, 2020), and ggplot2 v.3.3.3 (Wickham, 2016). The principal components (PCs) with eigenvalues larger than the average of all the eigenvalues (Kaiser-Guttman stopping rule) were retained (Jackson, 1993). The coordinates of the individuals in these components were used as input in a two-way ANOVA to assess the significance of fertilization and termination method on each component, with Tukey’s test (*α*=0.05) for *post hoc* multiple comparison of means with the emmeans package (Lenth, 2021).

In a complementary multivariate analysis, the generalized UniFrac distance and the Aitchison distance (i.e., Euclidean distance of clr-transformed data) were used as input in PERMANOVA (*α*=0.05; Anderson, 2001) with the *adonis* function (1,000 permutations) of vegan package v2.5–7 (Oksanen et al., 2020) to study the effects of the termination method, fertilization, and their interaction on community structure. When necessary, pairwise PERMANOVA were conducted with FDR *p*-value adjustment method in RVAideMemoire package v 0.9–80 (Hervé, 2021). The homogeneity of multivariate dispersions among treatments was visualized in vegan package v2.5–7 using *betadisper* function and a permutation test (*α*=0.05; 999 permutations).

## Results

### Quantitative PCR of *amoA*

A statistically significant main effect of the termination method (*p*<0.001) was detected for *amoA*_AOA_. Mowing resulted in a higher *amoA*_AOA_ abundance in the rhizosphere compared to control plants (1.49-fold change, *p*<0.01), and to those terminated with glyphosate (1.76-fold change, *p<* 0.001). No significant differences were observed for *amoA*_AOA_ between glyphosate termination and the untreated control (*p*>0.05; Table 1, Supplementary Figure S1). For the N fertilizer, a marginally significant main effect was observed (*p=* 0.06) with a higher copy number of *amoA*_AOA_ detected in the rhizosphere of unfertilized plants relative to the fertilized plants (1.14-fold change, Table 1).

**Table 1:** Quantitative PCR of *amoA* genes.

		AOB	AOA
Fertilization	*n*	Mean	Mean
Fertilizer (N)	9	6.12±0.08	5.91±0.05
No fertilizer	9	5.79±0.04	5.98±0.04
Termination method	n	Mean	Mean^**^
Glyphosate (G)	6	5.86±0.03	5.84±0.03^a^
Mowing (M)	6	6.07±0.10	6.08±0.03^b^
Untreated control (U)	6	5.94±0.14	5.91±0.03^a^
Fertilization×Termination method	n	Mean^*^	Mean
GN	3	5.89±0.04^bB^	5.77±0.03
MN	3	6.26±0.12^aA^	6.06±0.04
UN	3	6.21±0.14^aA^	5.9±0.07
G	3	5.83±0.04^bB^	5.9±0.01
M	3	5.88±0.03^bB^	6.11±0.04
U	3	5.67±0.09^bB^	5.92±0.02
	df	*p*-values (ANOVA)	
Fertilization	1	0.0006	0.063
Termination method	2	0.091	**0.0001**
Fertilization × Termination method	2	0.053	0.36

*The values are the mean of log_10_ copy number±SEM. M, mowing; G, glyphosate; U, untreated control. Letter N indicates N fertilization*.

* *Different upper case letters indicate statistically significant differences among termination methods (p<0.05) within each level of fertilization. Different lower case letters indicate statistically significant differences between the fertilized and unfertilized treatment within each termination method (p<0.05; significant interaction)*.

** *Different letters indicate statistically significant differences (p<0.05) among termination methods averaged through all levels of fertilization*.

*ANOVA p-values in bold indicate statistical significance (α = 0.05)*.

A differential response of *amoA*_AOB_ to the addition of N fertilizer was observed in the rhizosphere of glyphosate-treated plants relative to the response observed in mowed or in untreated plants (*p=* 0.053 for the interaction term). While no N-induced stimulation of AOB was observed in glyphosate-treated plants, a significantly greater *amoA* copy number was observed after N fertilization in the rhizosphere of mowed and control plants relative to the unfertilized plants (*p<* 0.05; lower case letters, Table 1; Supplementary Figure S1). A 2.5 and 3.6-fold change was observed after fertilization in mowed and control plants, respectively (Table 1). When comparing among termination methods, a significantly lower abundance of *amoA*_AOB_ was observed for glyphosate termination relative to both mowing and control within the fertilized condition (Table 1, upper case letters), a result that was not observed within the unfertilized condition.

### Potential Nitrification Activity

A statistically significant main effect of N fertilization was observed for PNA (*p*<0.001). A greater ammonia oxidation activity was observed in N fertilized treatments, regardless of the termination method applied (Table 2). In contrast, termination method had no influence on this functional assay (*p*>0.05). Further, these results were consistent with the finding from the addition of root exudates from glyphosate-treated plants or mowed plants to soil slurries. The addition of exudates did not show a significant effect in the nitrifying response measured by PNA (*p>* 0.05; Supplementary Figure S2).

**Table 2:** Potential nitrification activity (PNA).

PNA (μgN-NO_2_^−^ g^−1^ dws h^−1^)							
Mowing (M)		Glyphosate (G)		Untreated (U)		Fertilized	Unfertilized
M	MN	G	GN	U	UN		
1.81±0.05	3.61±0.36	2.1±0.08	3.66±0.39	2.15±0.1	3.41±0.07	***3.56±0.23*** ^**a**^	***2.02±0.5*** ^**b**^
**2.71±0.42**		**2.88±0.39**		**2.88±0.39**			

*Values in bold and italics indicate the mean of fertilized and unfertilized plants across termination methods (significant differences shown by different letters, p<0.05). Values in bold indicate the mean of each termination method across all fertilization levels. No statistically significant differences were detected among termination methods. The SEM is indicated in each case. Letter N indicates N fertilization*.

When analyzing abundance-activity correlations, a strong positive correlation was observed between PNA and *amoA*_AOB_ copies (*r*=0.78, *p<* 0.001), while no significant correlation was observed between *amoA*_AOA_ copy number and PNA showing a negative and weak correlation (*r*=−0.19, *p>* 0.05).

### Amplicon Sequencing

The total number of *amoA*_AOA_ and *amoA*_AOB_ sequences in each sample after each step (filtering, denoising, and chimera removal) is shown in Supplementary Tables S2, S3, respectively. As shown in these tables, each step was conducted without removing a large number of reads, reflecting the high quality of the datasets. For AOA, a total of 216 ASVs were detected with a final length of 283bp. A total of 113 ASVs were obtained for AOB with a final length of 452nt. The *amoA*_AOA_ and *amoA*_AOB_ amplicon sequencing datasets have been deposited in Sequence Read Archive (SRA) repository under the accession PRJNA701453. Rarefaction curves (Supplementary Figure S3) indicated that sequencing depth was enough to cover the full diversity of AOA and AOB, even at the rarefaction value (4,543 for AOA and 2,971 for AOB).

After clustering ASVs at the appropriate *amoA* identity threshold (90%), we obtained OTU tables for AOA (15 OTUs) and AOB (6 OTUs) with a considerably lower sparsity than the zero-enriched ASV tables, rendering them appropriate for further beta diversity analyses with multivariate methods and compositional data. The BLASTn search using the nucleotide database reported significant hits with *amoA* sequences of uncultured AOA (Supplementary Table S4) and uncultured AOB (Supplementary Table S5).

### Alpha Diversity

For AOA, no significant differences were observed for any of the alpha-diversity metrics (*p>* 0.05) between N-fertilized and unfertilized plants or among termination methods (Supplementary Table S6). Statistically significant greater values of alpha diversity metrics were observed for AOB in the rhizosphere of N fertilized plants compared to the unfertilized (*p=* 0.003 for Shannon diversity index H′ and *p=* 0.013 for the observed richness S′), except for Pielou’s evenness index (J’; *p=* 0.19). Conversely, no significant differences among termination methods were observed for any of the parameters studied (*p>* 0.05, Supplementary Table S7).

### Beta Diversity and Phylogenetic Analysis

**Figure 1:**
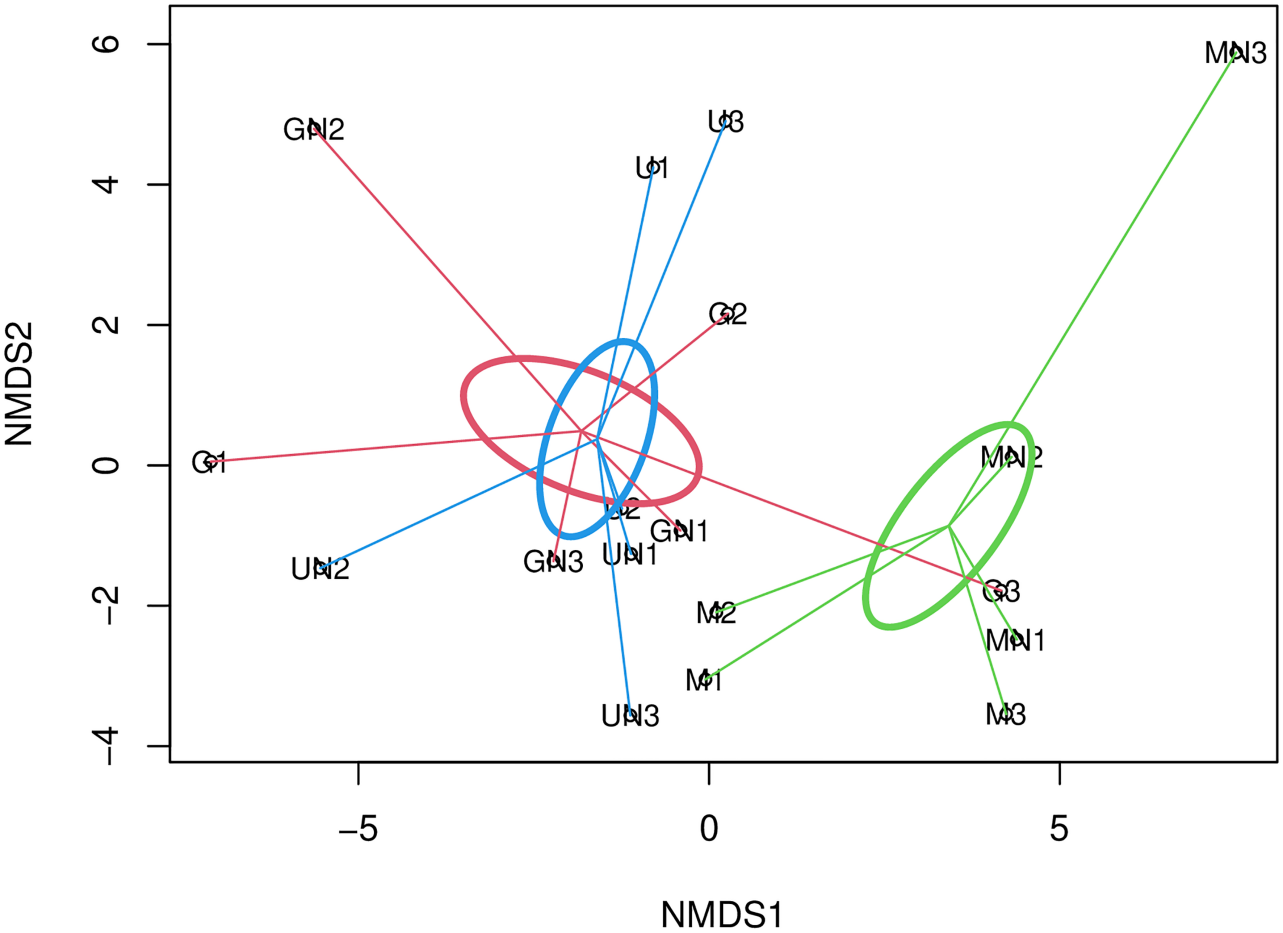
Non-metric multidimensional scaling (NMDS) using Aitchison distance (Stress=0.14, *R*^2^=0.98) for AOA. The SE of mowing termination, glyphosate termination, and untreated control is indicated with green, red and blue ellipses, respectively. The letter M in the labels of samples indicates mowing termination, letter G desiccation with glyphosate and letter U untreated control. Letter N indicates N fertilization and the number identifies the replicate of each treatment.

The multivariate statistical analysis through PERMANOVA to investigate the beta diversity of AOA indicated no interaction between fertilization and the termination method (Table 3). A marginally significant effect of fertilization was detected on AOA communities when the dataset was analyzed under the standard approach with the phylogenetic distance (UniFrac), while no significant effect of fertilization was observed with Aitchison distance (*p>* 0.05, Table 3). Contrary to AOB, a statistically significant effect of the termination method was observed on the community structure of AOA (*p<* 0.05, Table 3) for both Aitchison distance and the UniFrac distance. Pairwise PERMANOVA indicated significant differences between communities of glyphosate-treated and mowed plants (FDR adjusted *p=* 0.04) or between mowed plants and the untreated control (FDR adjusted *p=* 0.04). No significant differences were detected between glyphosate and the untreated control (FDR adjusted *p>* 0.05), as visualized in NMDS analyses based on Aitchison distance (Figure 1) and generalized UniFrac distance (Supplementary Figure S4).

**Table 3:** Beta-diversity of ammonia-oxidizing bacteria (AOB) and ammonia-oxidizing archaea (AOA) communities.

Term	Generalized UniFrac distance				Aitchison distance			
	df	*R* ^2^	F.model	*p*-value	df	*R* ^2^	F.model	*p*-value
**AOB**								
Termination method	2	0.082	0.783	0.624	2	0.1	0.838	0.551
Fertilization	1	0.192	3.673	**0.009**	1	0.13	2.17	0.089
Interaction	2	0.097	0.921	0.506	2	0.054	0.45	0.876
Residuals	12				12			
**AOA**								
Termination method	2	0.207	2.079	**0.027**	2	0.196	1.867	**0.037**
Fertilization	1	0.098	1.981	0.077	1	0.0386	0.733	0.617
Interaction	2	0.098	0.991	0.47	2	0.134	1.272	0.234
Residuals	12				12			

*results of PERMANOVA with 1,000 permutations are indicated. p-values in bold indicate statistical significance (α=0.05)*.

**Figure 2:**
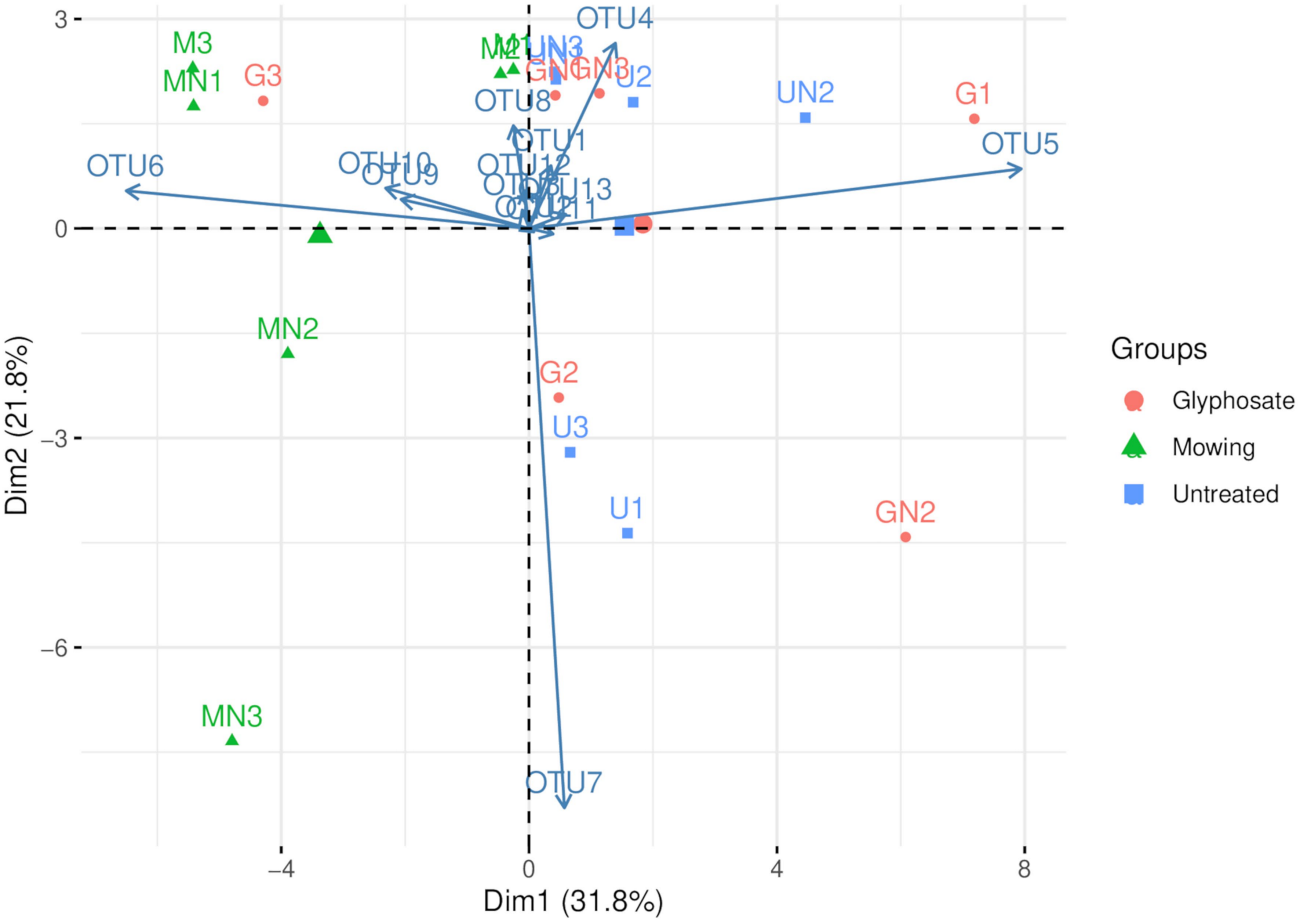
Compositional biplot of AOA communities using centered log-ratio transformed data (Aitchison transformation). The letter M in the labels of samples indicates mowing termination, letter G desiccation with glyphosate and letter U untreated control. Letter N indicates N fertilization and the number identifies the replicate of each treatment.

**Figure 3:**
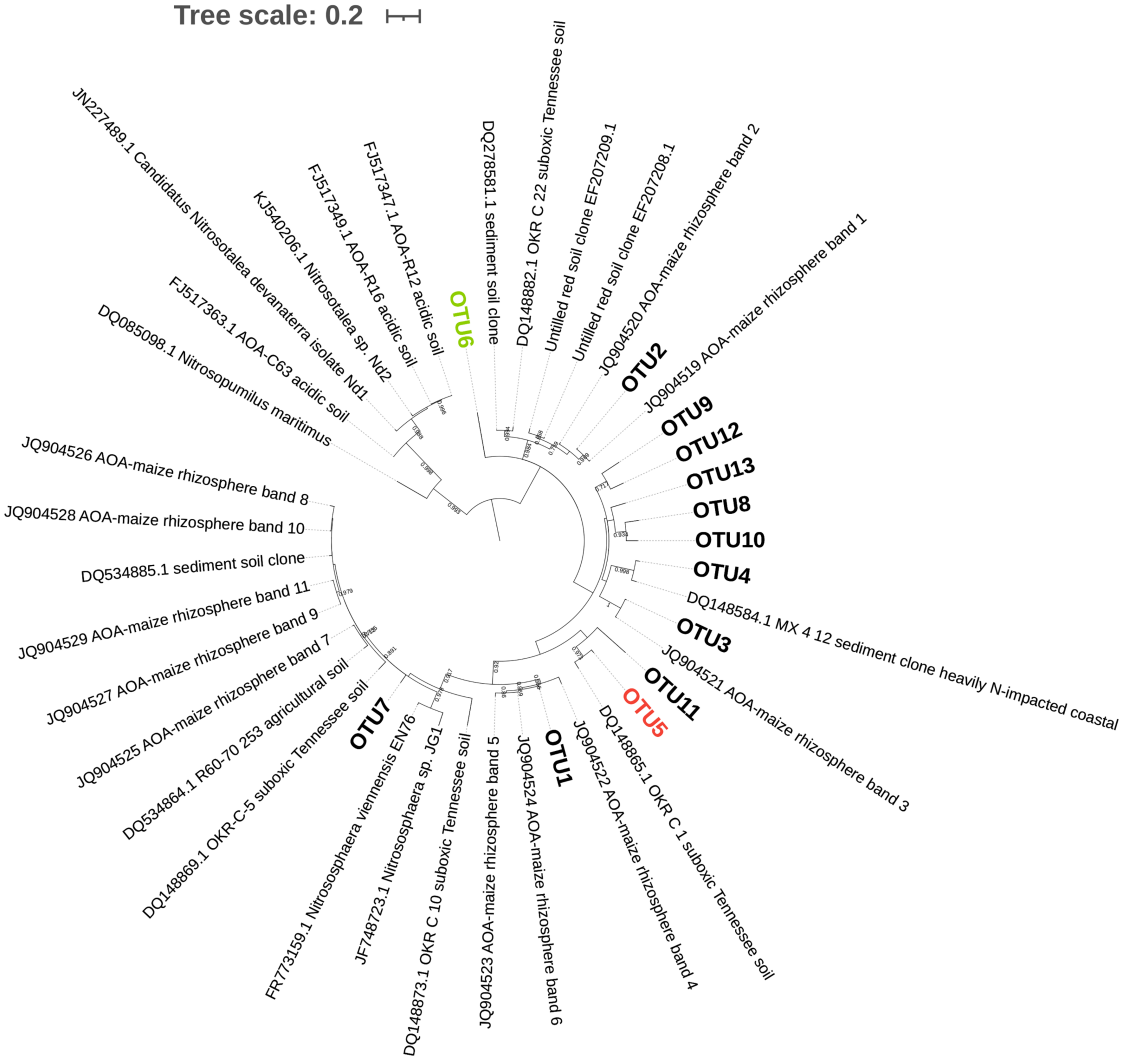
Phylogenetic tree of AOA with the 13 operative taxonomic units (OTUs) obtained after clustering amplicon sequence variants (ASVs) at 90% identity threshold. Green and red labels indicate OTUs highly correlated with PC1 in Figure 2. The evolutionary history was inferred by using the Maximum Likelihood method based on the Tamura 3-parameter model (lowest Bayesian Information Criterion index, BIC) in MEGAX. The tree with the highest log likelihood (−3195.09) is shown. Bootstrap values (>65%) are shown at branch nodes. A discrete Gamma distribution was used [five categories (+G, parameter=0.9061)]. The rate variation model allowed for some sites to be evolutionarily invariable [(+I), 46.46% sites]. The tree is drawn to scale, with branch lengths measured in the number of substitutions per site. The analysis involved 42 nucleotide sequences, including 13 OTUs and 29 database sequences. All positions with less than 95% site coverage were eliminated (partial deletion option). There were a total of 261 positions in the final dataset. Interactive Tree of Life (iTOL) tool was used for tree visualization.

Principal component analysis of AOA (Figure 2) showed that the first two PCs explained 53.5% of the total variance of the dataset. A separation across PC1 was observed between communities of mowed plants and the other treatments (Figure 2), as previously observed in NMDS analyses (Figure 1). The two-way ANOVA with PCs as variables indicated no significant interaction and a statistically significant effect of termination method on PC1 (*p<* 0.05, Table 4). Pairwise comparisons revealed no differences between glyphosate termination and the untreated control, while mowing termination was significantly different from both treatments (*p<* 0.05, Tukey’s HSD test). While an interaction between fertilization and termination method was observed for PC2 (Table 4), no significant differences were detected between fertilized and unfertilized samples within each termination method, nor among termination methods within fertilized or unfertilized samples (*p>* 0.05, Tukey’s HSD test). The contributions of OTU5 and OTU6 to the ordination on PC1 were notably greater than the contributions of the other OTUs (53.5 and 35.8%, respectively). A positive correlation was observed between OTU5 and PC1, while a negative correlation with this component was detected for OTU6 (Table 4). As shown in the biplot, OTU5 was enriched in communities from glyphosate-treated or control plants and depleted in the rhizosphere of mowed plants, the opposite trend was observed for OTU6 (Figure 2). Further phylogenetic analyses (maximum likelihood tree, Figure 3) indicated that the sequence of responsive OTU5 is related to the *amoA* sequence of an uncultured AOA isolated from a suboxic soil (red label, Figure 3). No clustering with the other OTUs in this study or with *amoA* sequences from maize rhizosphere, bulk soil, or sediments was observed for OTU6 (green label, Figure 3).

**Table 4:** Results of principal component analysis (PCA) for *amoA*_AOA_ centered log-ratio transformed data.

	Correlations			
	PC1	PC2	PC3	PC4
OTU1	0.18	0.46	0.46	−0.39
OTU2	0.03	−0.02	0.63	0.02
OTU3	−0.05	0.13	0.65	−0.33
OTU4	0.29	0.54	0.06	−0.55
OTU5	0.90	0.10	−0.19	0.37
OTU6	−0.83	0.07	−0.05	0.53
OTU7	0.07	−0.99	0.02	−0.07
OTU8	−0.06	0.35	0.31	−0.08
OTU9	−0.29	0.06	−0.90	−0.29
OTU10	−0.51	0.13	0.34	−0.09
OTU11	0.10	−0.02	−0.30	0.20
OTU12	−0.02	0.16	0.24	0.05
OTU13	0.20	0.07	0.62	−0.35
**Eigenvalues**				
	PC1	PC2	PC3	PC4
Eigenvalue	12.89	8.82	6.34	4.68
% variance	31.77	21.75	15.63	11.54
Cumulative % variance	31.77	53.52	69.16	80.70
**ANOVA (*p*-values)**				
	PC1	PC2	PC3	PC4
Termination	**0.02**	0.99	0.12	0.81
Fertilization	0.86	0.75	0.1	0.74
Termination × Fertilization	0.51	0.07	0.39	0.62

*The first four principal components (PCs) which explained 80.7% of the variance of the dataset are shown. The probalilty values associated with the ANOVA (termination method, fertilization and their interaction) are shown below. Statistical significance is shown in bold (α=0.05)*.

**Figure 4:**
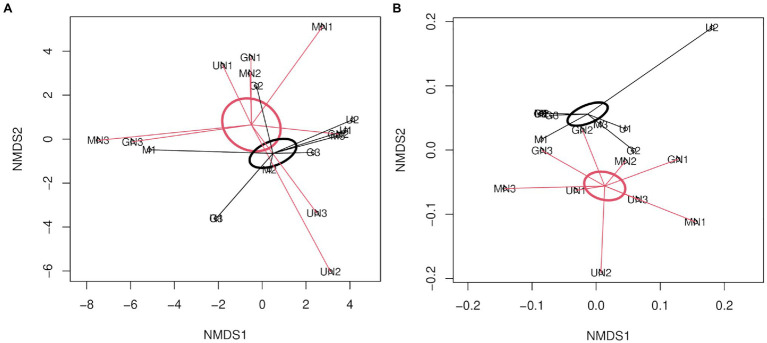
Non-metric multidimensional scaling of AOB using **(A)** Aitchison distance (Stress=0.13, *R*^2^=0.98; **B**) generalized UniFrac distance (Stress=0.08, *R*^2^=0.99). The SE of fertilized and unfertilized groups is indicated with red and black ellipses, respectively. The letter M in the labels of samples indicates mowing termination, letter G desiccation with glyphosate and letter U untreated control. Letter N indicates N fertilization and the number identifies the replicate of each treatment.

**Figure 5:**
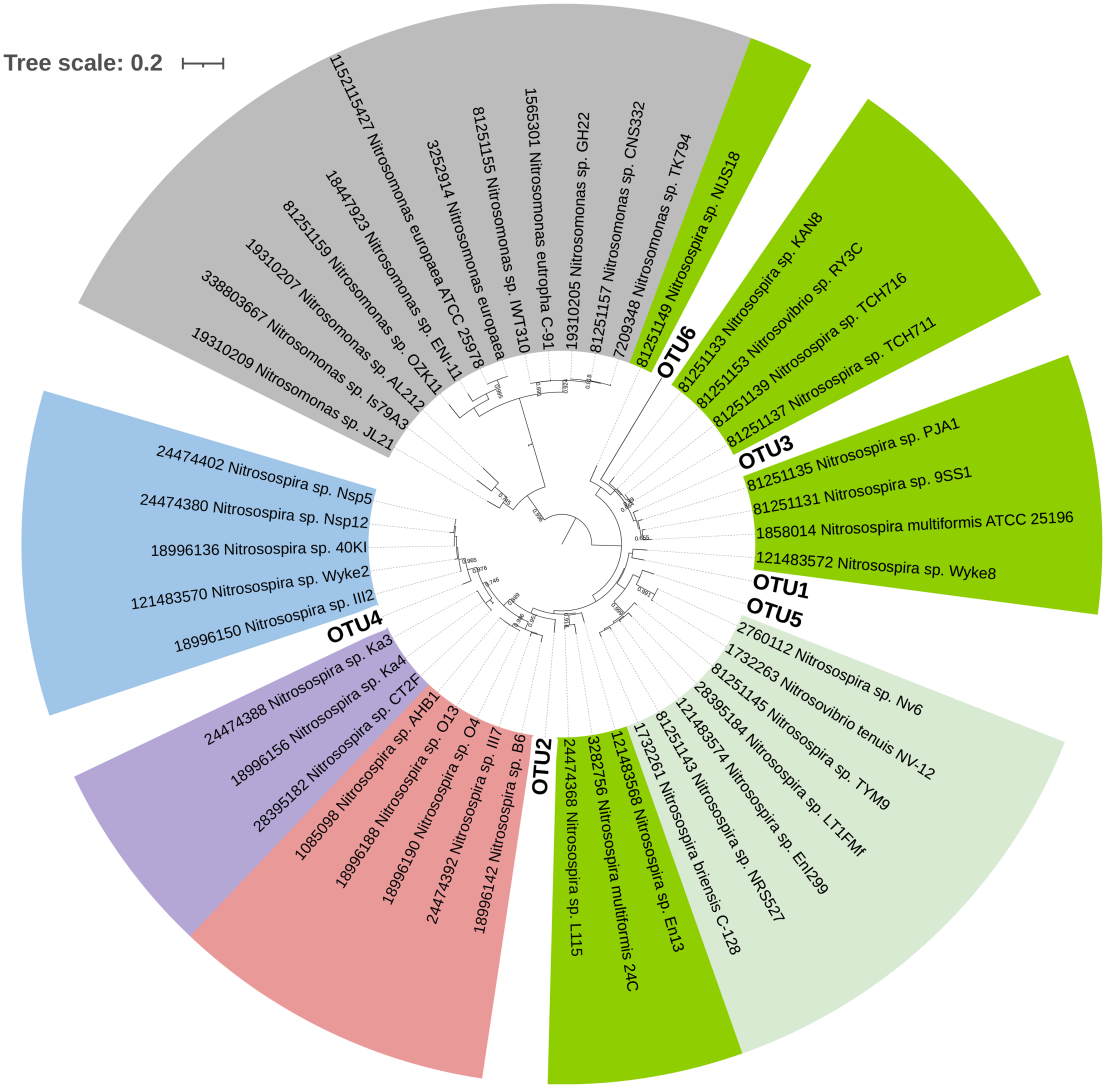
Phylogenetic tree of AOB with the six OTUs obtained in this study after clustering ASVs at 90% identity (bold letter). The different *Nitrosospira* clusters commonly found in soil are indicated in colours (light blue: cluster 0; light purple: cluster 4; light red: cluster 2; light green: cluster 3b and green: cluster 3a). *Nitrosomonas* lineage is shown in gray. The evolutionary history was inferred by using the Maximum Likelihood method based on Hasegawa-Kishino-Yano model (lowest Bayesian Information Criterion index, BIC) in MEGAX. The tree with the highest log likelihood (−4633.71) is shown. Bootstrap values (>65%) are shown at branch nodes. A discrete Gamma distribution was used [five categories (+G, parameter=0.2979)]. The tree is drawn to scale, with branch lengths measured in the number of substitutions per site. This analysis involved 50 nucleotide sequences. All positions with less than 95% site coverage were eliminated (partial deletion option). There were a total of 410 positions in the final dataset. iTOL tool was used for tree visualization.

For AOB, the statistical analysis of Aitchison and UniFrac distances through PERMANOVA indicated no interaction between fertilization and the termination method (*p>* 0.05, Table 3). N fertilization showed a marginally significant effect on the community structure of AOB for the Aitchison distance (Figure 4A) and a statistically significant effect for the phylogenetic distance (generalized UniFrac; Table 3 and Figure 4B). No significant effect of the termination method was detected on the community structure of AOB (PERMANOVA, *p>* 0.05, Table 3). The phylogenetic analysis of *amoA*_AOB_ amplicons (maximum likelihood tree) showed most OTUs within the *Nitrosospira* cluster 3 (green and light green labels, Figure 5) and only one OTU (OTU4) closer to *Nitrosospira* cluster 0 (light blue label, Figure 5). No *Nitrosomonas* spp. were detected in the rhizosphere of *A. sativa* plants grown in this soil.

## Discussion

In this study, we assessed the effects of two CC-associated practices (N fertilization and termination method) in the rhizosphere of *A. sativa* L. to elucidate how ammonia oxidation and the involved prokaryotic players (AOA and AOB) respond to both management strategies. This information is critical to further our understanding of the N dynamics in soils after CC termination and to comprehensively assess CC as a tool to foster agricultural sustainability.

Our results indicated that the objective of the study was achieved and the original hypotheses could be properly tested. We stated that the rhizosphere effects associated with mowed or glyphosate-terminated plants would exert a differential influence on prokaryotic ammonia oxidizers. The ammonia-oxidizing archaea responded mainly to the termination method (Table 3), as observed in the separation of communities of mowed plants from glyphosate terminated plants and the untreated control (Figure 1). Similarly, a significantly higher *amoA*_AOA_ gene abundance was detected in microbial communities of mowed plants (Table 1). These results agree with the first hypothesis and are supported by different studies on rhizospheric soil showing that only AOA are responsive to the rhizosphere effect and labile soil organic matter (Chen et al., 2008; Ai et al., 2013; Wattenburger et al., 2020) as well as by several studies that reported potential mixotrophic growth properties of AOA (Wessén et al., 2010; Mußmann et al., 2011; Tourna et al., 2011; Ai et al., 2013; Qin et al., 2014).

Glyphosate treatment in sensitive plants is known to stimulate the release of organic compounds on rhizodeposits through both a higher exudation of carbohydrates and amino acids (Kremer et al., 2005) as wells as through root-derived resources in dying roots (“green bridge” effect; Schlatter et al., 2017), providing organic compounds that could support mixotrophic growth of AOA. Even though this previous knowledge make us expect a higher abundance of AOA in the rhizosphere of desiccated plants, these results were not observed (Table 1). In contrast, our results suggest that the growth of AOA could be impeded after glyphosate termination, as opposed to the increase in abundance observed in the rhizosphere of mowed plants. This result is a meaningful outcome of the study and suggests that the expected increase in AOA abundance relative to the untreated control could be hampered by plant metabolites released after glyphosate-induced biochemical shifts. Based on these results and a significantly lower *amoA*_AOB_ abundance in the rhizosphere of glyphosate-treated plants (under the fertilized condition, Table 1), we acknowledge that further research is needed to elucidate whether glyphosate elicited the release of nitrification inhibitors. These inhibitors might have a more intense effect under the fertilized condition as active cells are more sensitive to environmental and external factors (Zhang et al., 2014; Zabaloy et al., 2017). Among them, we cannot exclude glyphosate itself, considering the exudation of this compound through the roots (Kremer et al., 2005) and the reported effects (inhibition) of glyphosate on *amoA* abundance (Zhang et al., 2018).

The compositional analysis of AOA communities through PCA revealed a separation between communities of mowed plants and the rest of the samples in PC1 (Figure 2, Table 3), with OTU5 and OTU6 as the most discriminant OTUs explaining the ordination (Table 4). We identified OTU5 as positively correlated with PC1 (Table 4) and, thus, more represented in the rhizosphere of glyphosate-treated plants (Figure 2). Contrary to OTU5, a negative correlation with PC1 was observed for OTU6, which was mainly represented in the rhizosphere of mowed plants. According to the maximum likelihood tree, OTU5 was phylogenetically close to an *amoA* sequence retrieved from an uncultured AOA of a saturated (suboxic) Tennessee soil (red label, Figure 3). This information suggests that OTU5 could be more adapted to lower oxygen levels in the rhizosphere of glyphosate-treated or untreated plants than in mowed plants. Further studies in the rhizosphere of terminated and control plants, including oximetry techniques, would be enlightening in this sense. On the other hand, OTU6 (green label, Figure 3) was more represented in the rhizosphere of mowed plants (Figure 2) and was found phylogenetically separated from all the other OTUs in the oat rhizosphere, from *amoA* sequences in the rhizosphere of maize and from bulk soil samples (Figure 3). The result could be indicating a strong link with the selection imposed by roots of mowed plants in the soil under study.

A marginally significant effect of fertilization was observed in AOA abundance, in an opposite way to AOB, i.e., fertilized plants showed a lower abundance of AOA relative to the unfertilized (Table 1). Similarly, the effect of fertilization in AOA community structure (Table 3) was weak (UniFrac distance) or non-significant (Aitchison distance), as opposed to the effect observed on AOB. Therefore, we confirmed the second hypothesis: AOB were the main responders to inorganic fertilizer addition. This result is supported by different studies showing that AOA prefer conditions, in which ammonium is released at continuous yet low rates through mineralization of organic matter (Stopnišek et al., 2010; Levičnik-Höfferle et al., 2012; Hink et al., 2018).

Under the fertilized condition, a significantly lower abundance of *amoA*_AOB_ was observed for glyphosate termination relative to the mechanical termination and the untreated control (Table 1, Supplementary Figure S1). Quantitative PCR also showed that the stimulative effect of N fertilization on *amoA*_AOB_ abundance depends on the termination method, as this effect was only observed in the rhizosphere of mowed and untreated control plants (Table 1, Supplementary Figure S1). This is a critical finding as it suggests that glyphosate termination counteracts the increase in AOB populations that would be normally induced by the inorganic N fertilizers in the rhizosphere or the bulk soil (Chu et al., 2008; Di et al., 2010; Glaser et al., 2010; Strauss et al., 2014). Further research is needed to elucidate the basis of such “repressive” glyphosate effect on the otherwise expected increase of AOB abundance after fertilization, including the potential release of inhibitors. In contrast, amplicon sequencing analysis of *amoA*_AOB_ revealed no differential effects of the termination method on the community structure (Table 3) or the alpha diversity metrics of AOB (Supplementary Table S7). Thus, for AOB in particular, we rejected our hypothesis regarding a differential influence of termination methods on this group, as termination effects depended upon the fertilization condition and were only observed on populations’ abundance.

In this study, a correlation was observed between PNA and *amoA*_AOB_. However, in glyphosate treated plants, the increase in PNA upon fertilization (Table 2) was not followed by an increase in *amoA*_AOB_ abundance levels (Table 1), as opposed to the response observed in mowed and control plants, suggesting that other important players in ammonia oxidation might be involed in the rhizosphere of glyphosate-terminated plants to explain the stimulation of PNA (Table 2). Moreover, PNA is inherently biased toward AOB rather than AOA (Ai et al., 2013) and thus, equal levels of PNA in the rhizosphere of glyphosate-treated plants, mowed and untreated plants (Table 2) might be matched by equal levels of *amoA*_AOB_, a result that was clearly not observed (Table 1). The lack of effects of root exudates on PNA (Supplementary Figure S2) might indicate that even if potential AOB-inhibitors would be acting on root exudates of glyphosate-treated plants (explaining the invariable levels of *amoA*_AOB_ upon fertilization), others nitrifiers should take a “back-up” function to maintain the same levels of ammonia oxidation that were observed in the rhizosphere of mowed and control plants. Among them, the AOA did not show a positive response to the fertilizer (Table 1). Thus, a potential role of heterotrophic nitrifiers and comammox *Nitrospira* in this “back-up” response should be investigated in the rhizosphere of fertilized CC after termination with glyphosate, where the availability of nutrients could be higher by a faster decay of roots (Imparato et al., 2016). Comammox *Nitrospira* from clades A and B have been both detected and quantitated in soil and in rice rhizosphere (Pjevac et al., 2017). Their role in nitrification in agricultural soils under CC-based managements deserves further research considering the detection of *Nitrospirae* members closely related to comammox *Nitrospira* in soil metegenomes, increasing their abundance upon fertilization (Orellana et al., 2018). Similalry, clade B of comammox *Nitrospira* has been linked to nitrification in contrasting soils when feeded with mineralized organic nitrogen (Wang et al., 2019). On the other hand, a second explanation would be an increase in *amoA*_AOB_ transcriptional activity upon fertilization even when *amoA* copy number did not change (Nicol et al., 2008). Further studies might help elucidate this disagreement between abundance and functional analyses in the rhizosphere of glyphosate-treated plants.

Our results demonstrated that, at the community structure level, AOB communities mainly responded to the fertilizer rather than to the termination method (Table 3). The results of beta diversity for *amoA*_AOB_ sequences indicated significant differences between communities from fertilized and unfertilized treatments, regardless of the termination method (Table 3). The effect of the inorganic fertilizer on AOB community structure in common oat rhizosphere is in agreement with the results reported by Ai et al. (2013) in maize rhizosphere and in barley rhizosphere (Glaser et al., 2010). At the taxonomic level, we found most OTUs in the *Nitrosospira* cluster 3 (green and light green labels, Figure 5), confirming that the dominant OTUs in soil belongs to *Nitrosospira* instead of *Nitrosomonas* (Fierer et al., 2009). Glaser et al. (2010) and Ai et al. (2013) also found most phylotypes in *Nitrosospira* cluster 3 for rhizospheric soil. In addition, we found one OTU (OTU4) closer to *Nitrosospira* cluster 0 strains (light blue label) than to cluster 2 (light red label), cluster 4 (light purple label) or cluster 3 strains (green and light green labels; Figure 5). Members of cluster 0 have been reported in soil, sand, and freshwater environments and showed high rates of nitrifier denitrification among members of *Nitrosospira* lineage (Shaw et al., 2006).

The consequences of niche specialization in the rhizosphere of terminated CC undoubtedly deserve more attention, considering that previous microcosm studies have reported a differential contribution to nitrous oxide emissions under high ammonium supply (inorganic fertilization) or low supply conditions (organic matter mineralization; Hink et al., 2018). Knowledge of the composition of ammonia oxidizer community can guide best fertilization strategies to optimize N use. A higher abundance of AOA and a different composition of this group of nitrifiers in roots of mowed plants could differentially influence the N availability for the cash crops, especially in soils receiving inorganic fertilizers. In AOA-dominated soils, the nitrification rate of ammonia derived from high inorganic fertilizer concentrations will be lower than in AOB-dominated soils (Hink et al., 2018), resulting probably in higher NH_4_^+^ levels for plant uptake after cash crop planting. Further field studies are required to test this hypothesis. The rhizosphere effects associated with the termination methods of a variety of CC other than oats, both alone or in mixture, could similarly influence nitrifying communities.

In conclusion, the results of this study indicate differential responses of AOA and AOB to fertilization and termination methods, reflecting a clear niche specialization in the rhizosphere of terminated oat plants. While AOB were the main responders to inorganic fertilizer addition due to their known preference for high ammonia levels, AOA were more sensitive to the termination method, probably due to a higher sensitivity of AOA to the specific rhizosphere effect of mechanically or chemically terminated plants. Termination with glyphosate counteracted the increase in AOB populations usually triggered by inorganic N fertilizers, showing a “repressive” effect not reflected at the functional level (PNA). A potential role of heterotrophic ammonia-oxidizers and comammox *Nitrospira* under glyphosate termination is suggested and proposed to be considered in future studies.

## Data Availability Statement

The datasets presented in this study can be found in online repositories. The names of the repository/repositories and accession number(s) can be found at: https://www.ncbi.nlm.nih.gov/sra, PRJNA701453.

## Author Contributions

MA and MCZ conceived and design the study. MA wrote the original draft, conducted the formal analysis and review, and performed PNA measurements. MEM and MCZ performed the greenhouse assay, soil sampling, DNA extraction, and qPCR. MCZ and MBV are credited for manuscript review, project administration, resources, and funding acquisition. MBV contributed to statistical analysis. All authors contributed to the article and approved the submitted version.

## Funding

This work was supported by the Argentinean National Agency for Scientific and Technological Promotion (ANPCyT) [grant number PICT 2015–1556]; the Universidad Nacional del Sur [grant number PGI 24/A240], the Office of International Programs – College of Agricultural, Consumer and Environmental Sciences (ACES), ACES International Seed Grant [grant number, ISGF2018-MV] and USDA grant ILLU-802-978. M. Allegrini and M.E. Morales hold postdoctoral and doctoral fellowships awarded by CONICET, respectively.

## Conflict of Interest

The authors declare that the research was conducted in the absence of any commercial or financial relationships that could be construed as a potential conflict of interest.

## Publisher’s Note

All claims expressed in this article are solely those of the authors and do not necessarily represent those of their affiliated organizations, or those of the publisher, the editors and the reviewers. Any product that may be evaluated in this article, or claim that may be made by its manufacturer, is not guaranteed or endorsed by the publisher.
